# Conducting osteochondral injury model in rabbit knee: Pearls and pitfalls

**DOI:** 10.1016/j.mex.2023.102323

**Published:** 2023-08-09

**Authors:** Arman Vahabi, Erdem Er, Semih Aydoğdu, Elcil Kaya Biçer

**Affiliations:** Department of Orthopaedics and Traumatology, Ege University School of Medicine, Izmir, Turkey

**Keywords:** Rodent, Animal experiment, Scaffold, Chondral damage, Osteochondral injury model in rabbit knee

## Abstract

Osteochondral damage is a commonly encountered issue in the daily orthopedic practice and has been extensively researched across various areas, including tissue transplantations, tissue engineering products, stem cell applications, and cell culture studies. The absence of a universally accepted treatment as the gold standard for osteochondral damage indicates the necessity for further studies in this field in the future. Although the biomechanical characteristics of the rabbit knee do not perfectly mimic those of the human knee, experimental studies conducted on rabbit knees are considered the most practical experimental model for testing a well-constructed experimental hypothesis. Our article endeavors to impart our practical insights and experiences to researchers without experience whom seeking to design studies utilizing this model. We aim to offer valuable guidance for preoperative, operative, and postoperative considerations.

• Rabbits used in osteochondral healing models should be at least 4 months old or older. Inducing damage in the trochlea is a well-established technique and relatively easy to apply.
• Do not use pointy ended drills as it might create uneven damage. Do not place applied treatment agent in inappropriate level in relation to the surrounding cartilage surface.

## Graphical abstract

**Figure:**
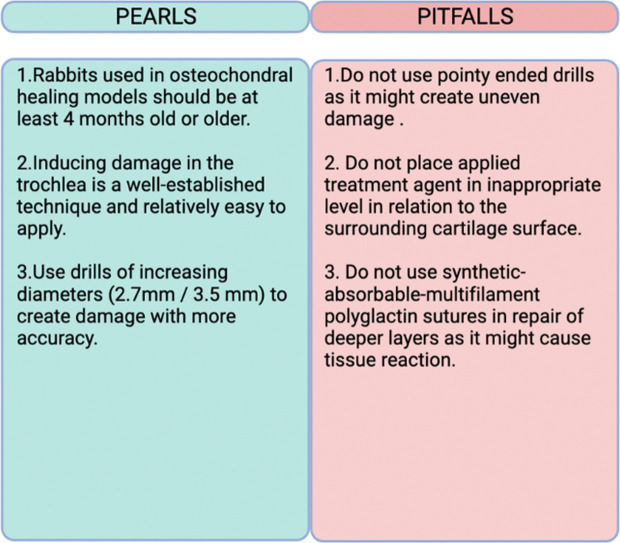

Specifications table

**Table:** 

Subject area:	Medicine and Dentistry
More specific subject area:	Osteochondral scaffolds
Name of your method:	Osteochondral injury model in rabbit knee
Name and reference of original method:	Rudert M. Histological evaluation of osteochondral defects: Consideration of animal models with emphasis on the rabbit, experimental setup, follow-up and applied methods. Cells Tissues Organs. 2002;171(4):229–40.
Resource availability:	https://www.arthrex.com/products/AR-1218–45
	https://www.dentalkart.com/

## Method details

### Study design

The age of the experimental animal plays a significant role, although it is often overlooked. It is widely recognized that wound healing is more efficient and rapid in young animals. Younger animals, particularly in relation to subchondral bone, have demonstrated superior healing capabilities. However, despite these advantages, satisfactory spontaneous repair of cartilage defects has not been achieved even in young rabbits [1]. Most researchers agree that rabbits reach adulthood between the ages of 4 to 6 months [2,3] However, some researchers argue that even at 6 months of age, epiphyseal plaques can still be observed on direct radiographs, suggesting that these rabbits should not be considered as adults [4]. Nonetheless, this distinction is not crucial for studies focusing on cartilage healing, as the repair capacity of cartilage remains suboptimal even in young animals. Based on these findings, it can be concluded that rabbits used in osteochondral healing models should be at least 4 months old or older.

The rabbit knee exhibits notable morphological similarities to the human knee joint in terms of bone geometry, cartilage, and tendons. However, certain differences exist. For instance, the longer patellofemoral groove in rabbits is likely attributed to their habitual crouching posture. Additionally, the anatomical structure of the extensor digitorum communis tendon in the rabbit knee differs from that in humans. It originates at the distal femur and passes over the lateral femoral condyle. There is considerable variation in the literature regarding the optimal location of the defect when creating osteochondral lesions in rabbits. Some authors have chosen to create defects in the patellofemoral groove to avoid postoperative load on injury site [5]. However, accessing the patellofemoral joint can be more challenging from a surgical technique perspective compared to the condyles. The condyles, on the other hand, have been frequently selected in studies due to their similarity to the weight-bearing surface of human joints [6]. Considering the unique anatomy of the rabbit knee, it is observed that the posteriormost parts of the condyles bear the load due to the squatting posture of rabbits. Additionally, the load distribution on the patellofemoral joint differs from that of humans due to the flexion position. These anatomical distinctions have led to a lack of consensus regarding the optimal location for creating defects in the rabbit model. Rudert reported that they did not observe a significant difference in the healing of osteochondral defects created in different locations [4]. We preferred to induce damage in the trochlea because it is a well-established technique and relatively easy to apply.

## Preoperative considerations

Pre-operative preparation begins with the provision of appropriate equipment and working environment. The animal should be anesthetized and shaved before being transported to the surgical table. Both intramuscular ketamine-xylazine combination and inhaled sevoflurane anesthesia are valid methods for anesthesia. Considering the ease of application and sufficient anesthesia time for the surgical procedure that will take approximately 20 min, we preferred ketamine-xylazine anesthesia in our applications.

For shaving, researchers have the flexibility to choose among different options such as hair removal cream, hair clipper, or razor, based on their personal preferences and available tool. However, based on our experience, we found that using a hair clipper to trim the hair and then shaving the surgical area with a razor blade is a practical and reasonable method. This approach facilitates the optimal preparation of the surgical site and allows for easy monitoring of the wound during the follow-up period.

While achieving optimal surgical site disinfection can be challenging during animal experiments, it is crucial to adhere to surgical antisepsis protocols as much as possible to minimize the risk of infection and potential complications that could impact the results. In this regard, we recommend taking precautions to reduce the risk of contamination at the surgical site. If there are concerns about complete decontamination of the foot, which may serve as a potential site for bacterial colonization, one approach is to cover the foot with a sterile glove or a sterile gauze pad. This additional measure helps to minimize the risk of contamination and maintain the integrity of the surgical site.

## Operative considerations

Following the stages of anesthesia, shaving, scrubbing, and covering, the surgical phase commences. The procedure begins with a 3 cm incision in the midline of the anterior of the knee. Upon passing through the skin and subcutaneous tissue, the joint capsule becomes visible. With the knee joint fully exposed, the arthrotomy is performed using the median parapatellar approach, ensuring that the repairable joint capsule remains in both sites of incision. Next, the intended area for damage is identified at the midpoint of the trochlea while the knee is in a semi-flexed position.

At this stage, a useful trick is to create a guiding entrance hole in the center of the intended damage area using a 0.5 mm burr commonly employed in dental procedures. Subsequently, drills of increasing diameters (2.7 mm / 3.5 mm) are utilized to enlarge the hole along the coronal axis. Finally, a 4.5 mm drill is employed to create the osteochondral damage, achieving the desired target size. While various applications may exist concerning the width and diameter of the desired damage, it is generally acknowledged that defects smaller than 3*3 *mm can heal spontaneously. Therefore, the objective is to create damages exceeding this threshold value. Taking into account the diameter of the scaffold to be used, we suggest applying a 3* *×* 5 mm scaffold by creating a 3 × 4.5 mm damage, which will enable a press-fit fixation.

There are two critical pitfalls to avoid. Firstly, it is essential to commence drilling with smaller drills before reaching the desired damage size. Skipping this step may result in uncontrolled deviations from the intended damage area and non-standardized damages due to potential oscillations of the drill in various directions. The second consideration pertains to achieving the intended depth of the damage. To address this, we recommend using blunt ended drill and marking the desired damage depth on the drill itself, thereby ensuring accurate and consistent depth during the procedure.

Following the creation of the defect, the subsequent step involves filling the defect in the treatment groups. Before applying the scaffold, graft, synthetic material, or treatment agent onto the defect, it is crucial to clean the defect area thoroughly. This can be achieved by using an injector to flush the area, ensuring that it is free from any particles or debris. This cleaning process helps to prepare the defect site for optimal interaction with the intended treatment material. An additional important consideration at this stage is the correct orientation of layered scaffolds. It is crucial to determine which side should be positioned on the bottom and which side should be placed on the top when placing the layered scaffolds. Typically, the layer that represents the bone layer should be positioned on the deeper side of the defect, while the layer representing the cartilage layer should be placed closer to the articular surface. This arrangement ensures proper integration of the scaffold with the surrounding tissues and supports the regeneration of both bone and cartilage components in the defect site.

Another important pitfall to avoid during the treatment phase is ensuring that the applied treatment agent remains at the appropriate level in relation to the surrounding cartilage surface after defect repair. It is crucial to maintain the same level as the surrounding cartilage tissue to maintain consistency in load distribution and achieve optimal standardization. If the treatment agent is positioned below or above the natural cartilage level, it can alter the loads on the defect area and potentially impact the results obtained. Therefore, careful attention should be given to ensure that the treatment agent used to fill the defect is positioned at the same level as the surrounding cartilage tissue, ensuring optimal standardization throughout the experiment.

The choice of suture material for wound closure is an important consideration. In the repair of the joint capsule, synthetic-absorbable-multifilament polyglactin sutures are not preferred due to their potential to cause tissue reaction (as we experienced such complication in earlier phases). Instead, monofilament absorbable sutures may be preferred for joint capsule repair. The same suture material used for joint capsule repair can also be utilized for repairing the subcutaneous layer. When it comes to skin sutures, our recommendation is to use non-absorbable monofilament synthetic sutures. Following the completion of suturing, the surgical incision is thoroughly cleansed with povidone iodine solution. No dressing is applied to the incision site. Subsequently, the animal is transferred to a designated postoperative recovery area, where measures are taken to ensure its protection from hypothermia and hypovolemia.

## Postoperative considerations

Amoxicillin-clavulanate is a recommended approach for surgical prophylaxis. Administering a single daily dose of amoxicillin for a period of 3 days is considered sufficient. We advise conducting daily follow-up for 2 weeks after the operation to monitor the wound site closely. For wound care, topical aerosol formulations containing tetracycline can be utilized on a daily basis. Alternatively, povidone iodine can also serve as a suitable alternative for wound management. It is important to note that rodents have a tendency to gnaw at sutures. Therefore, close monitoring of the wound site, particularly in the early postoperative period, is crucial. Based on our observations, even though animal gnaws the sutures after three days following surgery, the wound remains intact. However, during the first three days wound epithelization is not enough and, if the sutures are opened, it is necessary to re-suture the wound.

Another crucial parameter to consider is the duration of follow-up after the surgical procedure in rabbits. Some authors reported that the degeneration of osteochondral defects persisted even after 12 months while some achieved considerable healing within 12 weeks. Taking these into account, it can be concluded that the optimal follow-up period should be at least 6 months. However, it is worth noting that in the literature, many researchers still utilize a 12-week follow-up period despite the potential limitations in capturing long-term degenerative changes [7], [8], [9].

**Fig. 1:**
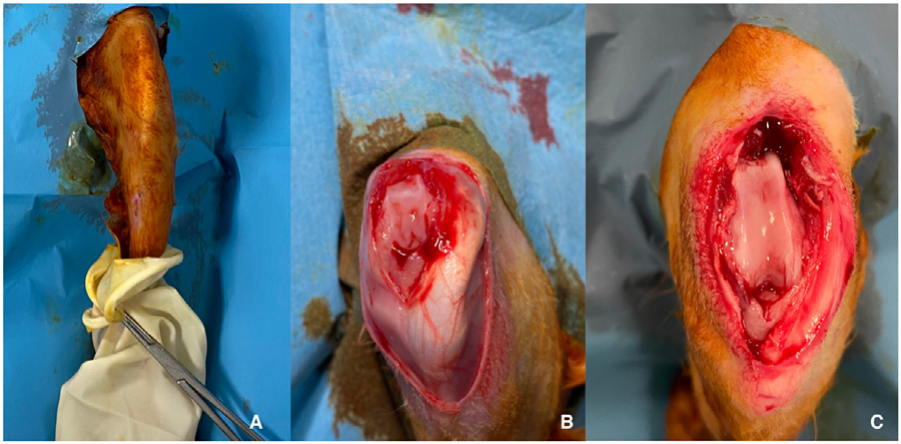
1A Sterile surgical glove used to reduce contamination risk. 1B: Approach to joint after median-parapatellar incision 1C Exposure of femoral trochlea following lateral deviation of extensor mechanism.

**Fig. 2:**
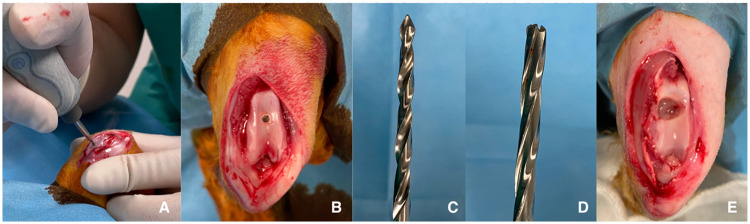
2A Marking intended damage area with 0.5 mm burr 2B: Marked area after initial damage created with burr 2C-D: Drills that grow in successive diameters, note that the drill to be used in the final step (4.5 mm) is chosen with a blunt tip so that it does not cause deeper damage in the center of created osteochondral defect. 2E Created osteochondral damage in its final form.

**Fig. 3:**
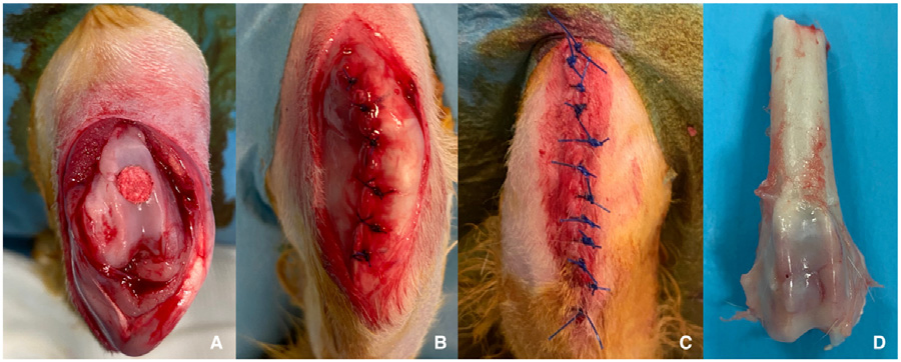
3A: Defect site repaired with osteochondral scaffold. 3B: Closure of joint capsule-extensor mechanism with monofilament absorbable sutures. 3C: Closure of skin with non-absorbable monofilament synthetic suture. 3D: Harvested sample for further analyses.

Following sacrifice, the next step is to collect tissue samples for analysis. For many research designs, sampling distal femur from 3 cm above the joint line in one piece is considered appropriate. However, the specific materials to be included in the sampling may vary depending on the research design. The knee joint and distal femur should carefully be separated from the surrounding joint capsule, muscle tissue, etc. and the sample is harvested by cutting the femur at the mid-shaft level (3 cm above the joint line) using a rongeur or, if available, a micro-oscillating cutter motor. This allows for the retrieval of the desired bone sample for further analysis (Fig. 1, Fig. 2, Fig. 3)

## Ethics statements

We confirming that those experiments complied with the ARRIVE guidelines and were carried out in accordance with the U.K. Animals (Scientific Procedures) Act, 1986 and associated guidelines; EU Directive 2010/63/EU for animal experiments; or the National Institutes of Health guide for the care and use of laboratory animals (NIH Publications No. 8023, revised 1978).

Animals are included to study regardless of their gender. It was not predicted that the variables we studied in our projects would be affected by sex hormones.

## CRediT authorship contribution statement

**Arman Vahabi:** Conceptualization, Methodology, Writing – original draft. **Erdem Er:** Conceptualization, Methodology, Writing – original draft. **Semih Aydoğdu:** Supervision, Writing – review & editing. **Elcil Kaya Biçer:** Supervision, Writing – review & editing.

## Declaration of Competing Interest

The authors declare that they have no known competing financial interests or personal relationships that could have appeared to influence the work reported in this paper.

## Data Availability

No data was used for the research described in the article.
